# Fabrication of Gold Nanoparticles/Graphene-PDDA Nanohybrids for Bio-detection by SERS Nanotechnology

**DOI:** 10.1186/s11671-015-1101-2

**Published:** 2015-10-12

**Authors:** Andreas H H Mevold, Wei-Wu Hsu, Andri Hardiansyah, Li-Ying Huang, Ming-Chien Yang, Ting-Yu Liu, Tzu-Yi Chan, Kuan-Syun Wang, Yu-An Su, Ru-Jong Jeng, Juen-Kai Wang, Yuh-Lin Wang

**Affiliations:** Department of Materials Science and Engineering, National Taiwan University of Science and Technology, Taipei, 106 Taiwan; Department of Materials Engineering, Ming Chi University of Technology, New Taipei City, 24301 Taiwan; Institute of Polymer Science and Engineering, National Taiwan University, Taipei, 106 Taiwan; Center for Condensed Matter Sciences, National Taiwan University, Taipei, 10617 Taiwan; Institute of Atomic and Molecular Sciences, Academia Sinica, Taipei, 10617 Taiwan; Department of Physics, National Taiwan University, Taipei, 10617 Taiwan

**Keywords:** Gold nanoparticles, Graphene, Surface-enhanced Raman scattering, Bio-detection

## Abstract

In this research, graphene nanosheets were functionalized with cationic poly (diallyldimethylammonium chloride) (PDDA) and citrate-capped gold nanoparticles (AuNPs) for surface-enhanced Raman scattering (SERS) bio-detection application. AuNPs were synthesized by the traditional citrate thermal reduction method and then adsorbed onto graphene-PDDA nanohybrid sheets with electrostatic interaction. The nanohybrids were subject to characterization including X-ray diffraction (XRD), transmission electron microscopy (TEM), zeta potential, and X-ray photoelectron spectroscopy (XPS). The results showed that the diameter of AuNPs is about 15–20 nm immobilized on the graphene-PDDA sheets, and the zeta potential of various AuNPs/graphene-PDDA ratio is 7.7–38.4 mV. Furthermore, the resulting nanohybrids of AuNPs/graphene-PDDA were used for SERS detection of small molecules (adenine) and microorganisms (*Staphylococcus aureus*), by varying the ratios between AuNPs and graphene-PDDA. AuNPs/graphene-PDDA in the ratio of AuNPs/graphene-PDDA = 4:1 exhibited the strongest SERS signal in SERS detection of adenine and *S. aureus*. Thus, it is promising in the application of rapid and label-free bio-detection of bacteria or tumor cells.

## Background

Raman scattering was discovered by CV Raman in 1928, and further, the use of surface-enhanced Raman scattering (SERS) technology was developed by Fleischman and others in 1974. SERS is used widely in various applications such as label-free sensing of bacteria *Escherichia coli (E. coli)* and various molecules. This is possible because of the enhancement of the Raman signal. Gold and silver nanoparticles are widely used for SERS enhancement [1–3] via their localized surface plasma resonance (LSPR). LSPR can increase the intensity of the Raman signal by at least 10^9^, thereby easily detecting the presence of various bacteria or molecules. Once metal (gold and silver) become nanoparticles with a unique size and morphology, their optical, electrical, and magnetic properties also change. Recent research on SERS technology emphasize on controlling the size and morphology of the nanoparticles. When the gap of the metal nanoparticles is within 10 nm, it will produce “hot spot” effect, which will further enhance the intensity of the SERS signal. Therefore, it is important to develop the SERS bio-detection by controlling the gap and the particle size of the metals nanoparticles.

Graphene [4] is an allotrope of carbon in the form of a two-dimensional hexagonal lattice, with its sp2 hybridization and very thin atomic thickness (of 0.345 Nm). It is formed from a single layer of graphite structure. Graphene was successfully isolated in 2004 by physicist Novoselov and Geim from graphite [5]. What make graphene so unique are its remarkable strength, electricity, and heat conduction, as well as many others. Graphene has many very specific physical properties, such as (1) high mechanical strength, Young’s modulus can reach 1000 GPa [6]; (2) thermal conductivity that can reach 5300 W/mK, which is higher than metals or diamond [7]; (3) electron mobility that can exceed 200,000 cm^2^/Vs with a resistance value (10–6 Ω·cm) even lower than silver or copper [8], which is the least resistance value of any currently known materials. Since graphene has excellent electrical and thermal conductivity and physical properties, it can be widely applied in different fields and has become a very popular research topic in recent years [9–12].

**Fig. 1:**
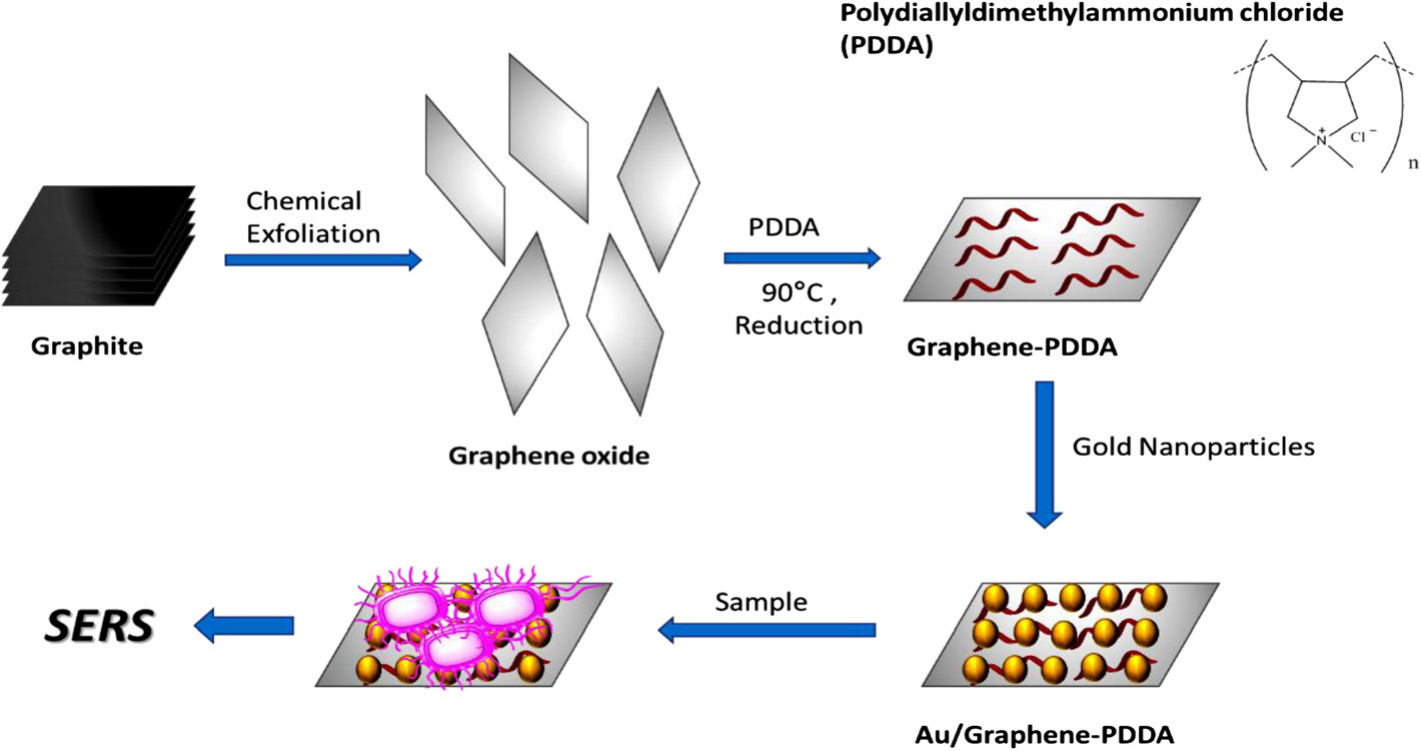
Fabrication of nanohybrids Au/graphene-PDDA

Poly(diallyldimethylammonium chloride) (PDDA) is a homopolymer and synthesized by George Butler in 1957 [13–14]. PDDA is a water-soluble polymer, and its structure is shown in Fig. 1. Its quaternary ammonium salt structure enables it to display a high charge density. PDDA thereby has cohesive, adsorption, and antibacterial properties, which is considered harmless for the human body. It is widely used in various applications, such as wastewater treatment plants and various biological and medical applications [15]. In 2005, Yang et al. [16] showed that PDDA can be adsorbed onto carbon nanotubes by non-covalent bonding or π-π interaction, in order to improve water dispersibility of nanotubes. The surface properties of nanotubes are similar to that of graphene, thereby PDDA can also adsorb onto graphene structure via their structural π-orbitals. After adsorption, the resulting surface charge of graphene is positive and prevents aggregation of graphene in water.

In this experiment, Au/graphene-PDDA nanocomposite was fabricated by adsorbed gold nanoparticles (AuNPs) onto the graphene-PDDA nanosheets as shown in Fig. 1. Graphite was a chemical exfoliated into graphene, PDDA was adsorbed onto graphene by reduction method, and AuNPs (negative charge) was then bonded onto the resulting graphene-PDDA nanosheets (positive charge) by ionic binding. Graphene-PDDA nanosheets are the supporting substrate to uniformly embed the AuNPs for creating more homogenous “hot spots” and controlling the interparticle gap of AuNPs. The positive charge of AuNPs/graphene-PDDA nanosheets is used to easily capture the negative charge of *Staphylococcus aureus* for SERS rapid detection. Various ratios of AuNPs/graphene-PDDA were evaluated in order to create an optimum surface-enhanced Raman scattering (SERS) signal for SERS bio-detection [17–20] of small molecules (adenine) and microorganisms *(S. aureus*).

## Methods

### Materials

The materials used in this study were as follows: graphite powder, <20 μm, synthetic, Aldrich; sulfuric acid, H_2_SO_4_, 96.5 %, Baker; fuming nitric acid, HNO_3_, ≧99.5 %, Sigma-Aldrich; potassium permanganate, KMnO_4_, Baker; PDDA, 35 % (average *M*_w_ < 100,000), Aldrich; hydrogen peroxide, H_2_O_2(aq)_, 35 %, Acros; hydrochloric acid, HCl_(aq)_, 37 %, Scharlau; sodium citrate dehydrate, Na_3_Ct·2H_2_O, ≧99.5, Sigma-Aldrich; hydrogen tetrachloroaurate(III) trihydrate, HAuCl_4_·3H_2_O, 99 %, Sigma-Aldrich; nitric acid, HNO_3(aq)_, 69 %, Panreac; silicon Oil, Choneye Pure Chemical; Luria-Bertani (LB broth), Difco™ (Agar Bacteriological), Oxoid; and adenine, C_5_H_5_N_5_, ≧99 %, Sigma.

### Synthesis of Gold Nanoparticles

Citrate thermal reduction method was used to prepare gold nanoparticles. It is an oxidation-reduction reaction, which uses sodium citrate (Na_3_Ct·2H_2_O) as a reducing agent to reduce Au^3+^ of HAuCl_4_·3H_2_O. The experimental procedure is as follows. (1) Ninety-six milliliters of 0.307 mM tetrachloroauric acid solution was prepared. (2) The solution was heated, and after boiling for 10 min, 4 mL of 1 % sodium citrate solution was added and the color changed from yellow to dark red.

### Synthesis of Graphene Oxide

Graphene oxide (GO) was prepared by using the modified Hummers method. Potassium permanganate was added to the graphite, and the surface of the graphite will have many oxidized functional groups. The experimental procedure is as follows. (1) Thirty-six milliliters of concentrated sulfuric acid was added to 1.0 g of graphite powder and stirred for 1 h. (2) The solution was stirred continuously in ice bath, 12 mL of fuming nitric acid was added dropwise, and then, 5 g of potassium permanganate was slowly added. (3) The solution was stirred for 120 h under room temperature. (4) One hundred twenty milliliters of deionized water was added slowly and stirred for 2 h under room temperature. (5) Six milliliters of hydrogen peroxide solution was added and stirred for 2 h and left at room temperature for 24 h. (6) The top layer of the clear solution was removed, and 200 mL of deionized water, 1 mL of hydrogen peroxide solution, and 1 mL of hydrochloric acid were added. The solution was mixed for 2 h and centrifuged. (7) Step 6 was repeated three times. (8) The top layer was washed with deionized water until the solid pH is close to 7. (9) After the solid was collected, it was placed in a vacuum oven and dried at 40 °C for 48 h. The final product was graphene oxide powder.

### Synthesis of Graphene-PDDA

The experimental procedure is as follows. (1) Sixty milligrams of graphene oxide powder was mixed with 20 mL of deionized water. (2) The solution was sonicated for 10 min. (3) Eight hundred microliters of PDDA was added and stirred for 10 min. (4) The solution was heated to 90 °C under reflux for 12 h. (5) The solution was centrifuged, and the upper layer solution was removed. The step was repeated for several times. (6) Deionized water was added to the final product.

### Synthesis of Au/Graphene-PDDA

Different proportions of graphene-PDDA and HAuCl_4_ solutions were prepared as shown in Table 1. Various ratios of HAuCl_4_ to graphene-PDDA were as follows: 1:2, 2:1, 4:1, 8:1, 16:1, referred as Au1/G2, Au2/G1, Au4/G1, Au8/G1, and Au16/G1.

**Table 1:** Various ratios of AuNPs to graphene-PDDA

Graphene-PDDA (3 mg/mL) μL	HAuCl_4_ (0.1 mg/mL) mL	DI water μL	Au/G (*w*/*w*)
333	5	17	1/2 (Au1/G2)
83	5	267	2/1 (Au2/G1)
42	5	308	4/1 (Au4/G1)
21	5	329	8/1 (Au8/G1)
10.5	5	339.5	16/1 (Au16/G1)
0	5	350	Au

### SERS Measurements by AuNPs/Graphene-PDDA Nanohybrids

A Raman microscope (HR800, Horiba, Japan) with He-Ne laser (632.8 nm) was used to detect the presence of *S. aureus* (ATCC 6538P). The experimental procedure is as follows. (1) Fifty microliters of the varied AuNPs/graphene-PDDA and 50 μL of *S. aureus* solutions (1 × 10^5^ CFU/mL grown for 18 h at 37 °C) or adenine (concentration of adenine is 10^−4^ M) were placed in 1.5 mL micro-centrifuge tubes and mixed well. (2) Five microliters of each sample was dropped on the aluminum sheet. Raman spectra in the range of 400 to 1800 cm^−1^ were evaluated for the samples. The intensity of the Raman signal at 733 cm^−1^ (SERS signal from the cell wall of *S. aureus*) was investigated also for the samples.

### Characterization Analysis of AuNPs/Graphene-PDDA Nanohybrids

The interaction between AuNPs and graphene-PDDA were analyzed by X-ray photoelectron spectroscope (XPS, VG ESCA Scientific, Theta Probe), and surface electric properties of AuNPs/graphene-PDDA samples were analyzed by zeta potential analyzer (Nano S90, Malvern Instruments) as described below.

## Results and Discussion

### Characteristics of Au/Graphene-PDDA

**Fig. 2:**
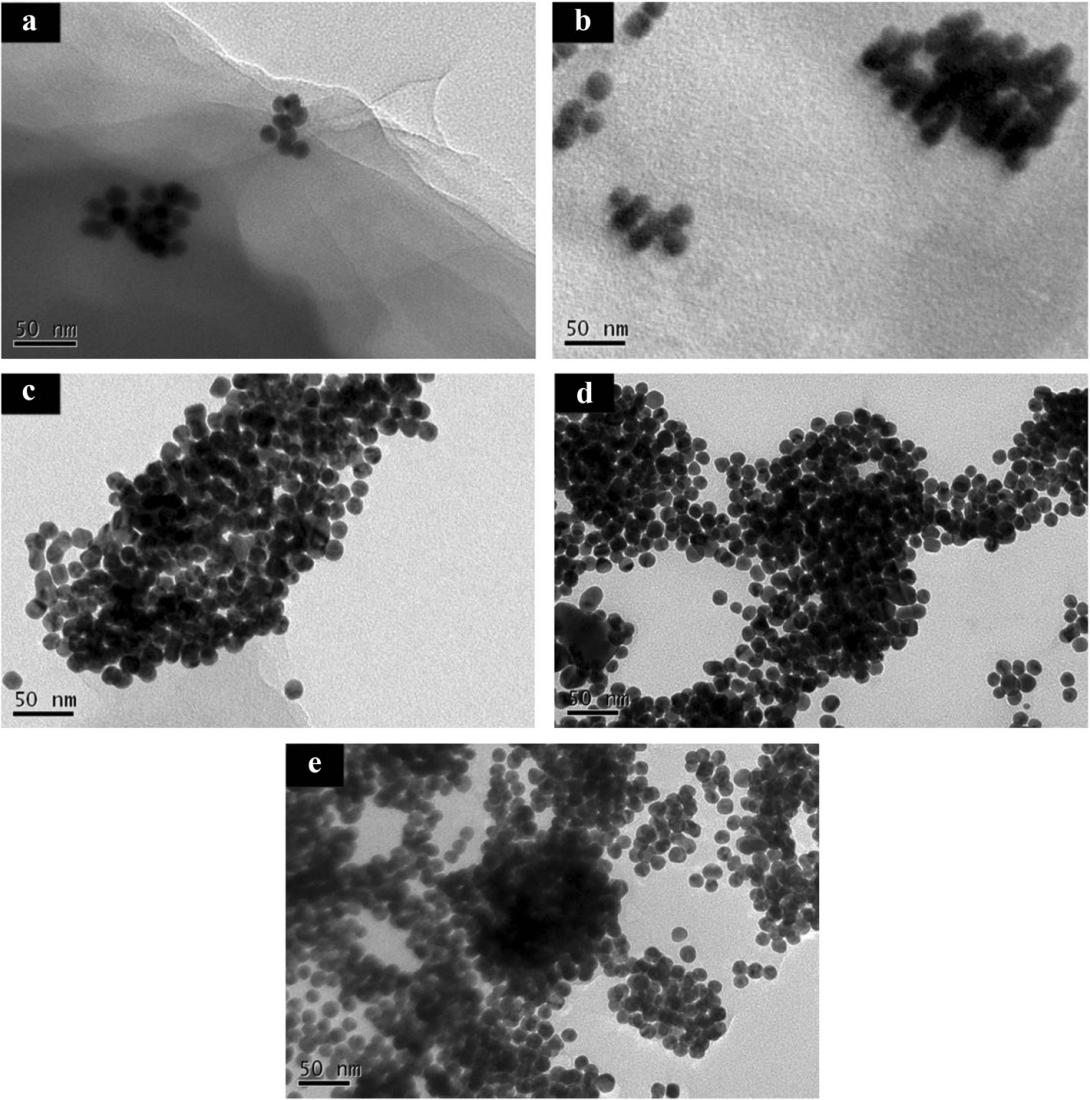
TEM of various ratios of Au/graphene-PDDA: **a** Au1/G2, **b** Au2/G1, **c** Au4/G1, **d** Au8/G1, and **e** Au16/G1

Various ratios of AuNPs to graphene-PDDA nanohybrids were prepared, including Au1/G2 (AuNPs to graphene-PDDA ratio = 1:2), Au2/G1, Au4/G1, Au8/G1, and Au16/G1. The morphology and distribution of the Au/graphene-PDDA nanohybrids were analyzed by transmission electron microscopy (TEM), as shown in Fig. 2. The results showed the diameter of AuNPs were about 15–20 nm, immobilized on the few layers of the graphene-PDDA sheets.

**Fig. 3:**
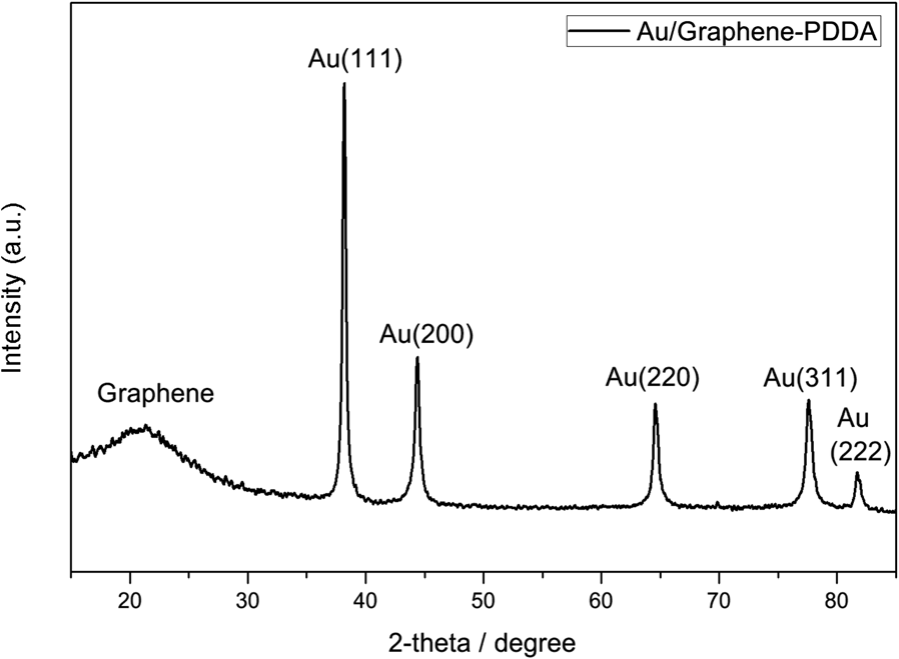
X-ray diffraction of Au/graphene-PDDA

X-ray diffraction of Au/graphene-PDDA (Fig. 3) shows that diffraction plane peaks of Au (111), Au (200), Au (220), Au (311), and Au (222), and corresponding to 2θ angle are 38.2°, 44.4°, 64.6°, 77.6°, and 81.7°, respectively, showing face-centered cubic (FCC) crystal structure. This is consistent with diffraction peak position of JCPDS database (Au, JCPDS file: 04-0784). It confirms that AuNPs are adsorbed onto the surface of graphene-PDDA nanosheets.

**Fig. 4:**
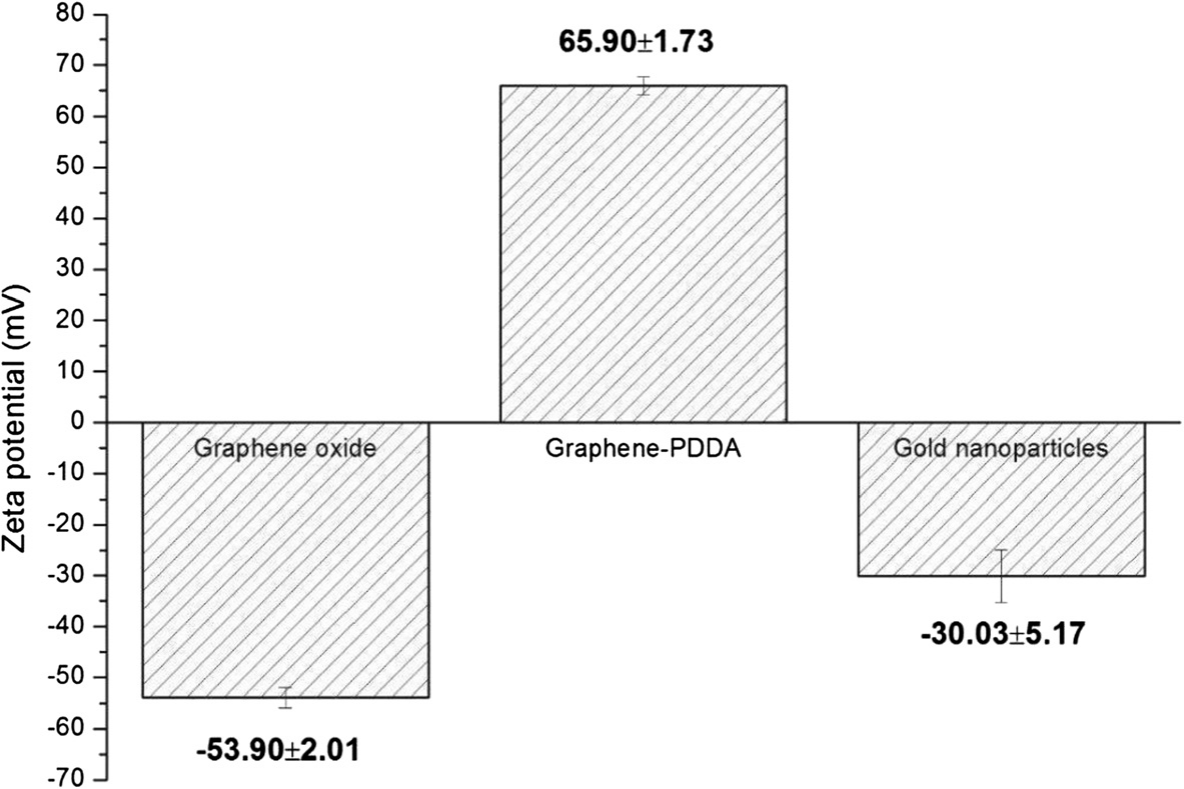
Zeta potential of graphene oxide, graphene-PDDA, and gold nanoparticles

**Fig. 5:**
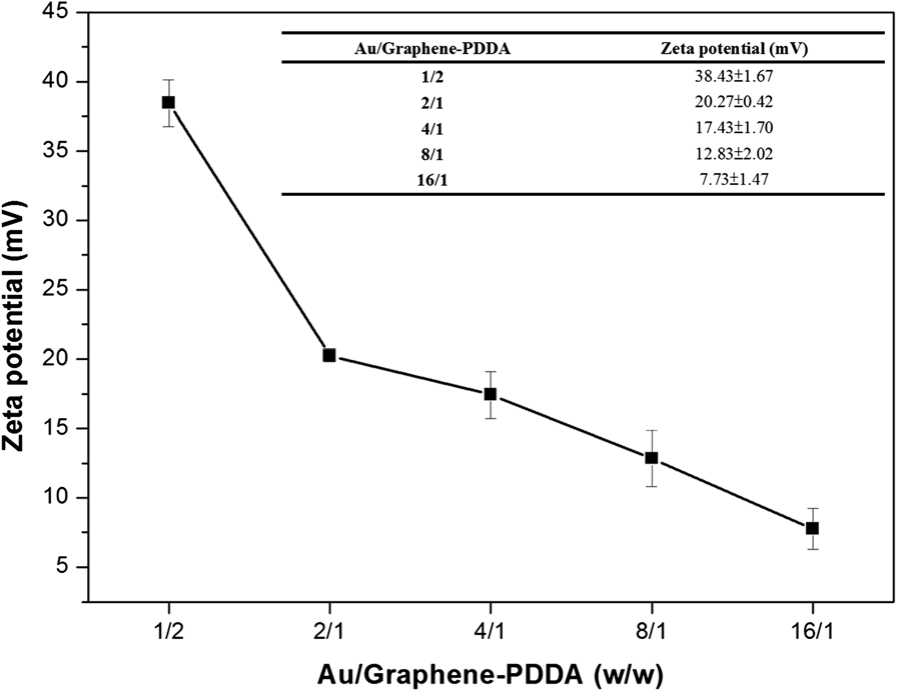
Zeta potential of various ratios of Au/graphene-PDDA

Zeta potential also confirmed successful fabrication of Au/graphene-PDDA, as shown in Fig. 4. The surface of graphene oxide is negative charge (−52.90 ± 2.01 mV), while graphene-PDDA surface is positive charge (65.90 ± 1.73 mV) due to the NH_2_ functional group in the PDDA, which enables it to attach to the negative charge of AuNPs. The higher addition of AuNPs would decrease the average zeta potential of the nanocomposites. In Fig. 5, it further confirms the successful preparation to add AuNPs onto graphene-PDDA nanosheets. With the increase in the ratios of AuNPs on the graphene-PDDA, zeta potential would decrease, proving that AuNPs are attached onto the surface of graphene-PDDA.

**Fig. 6:**
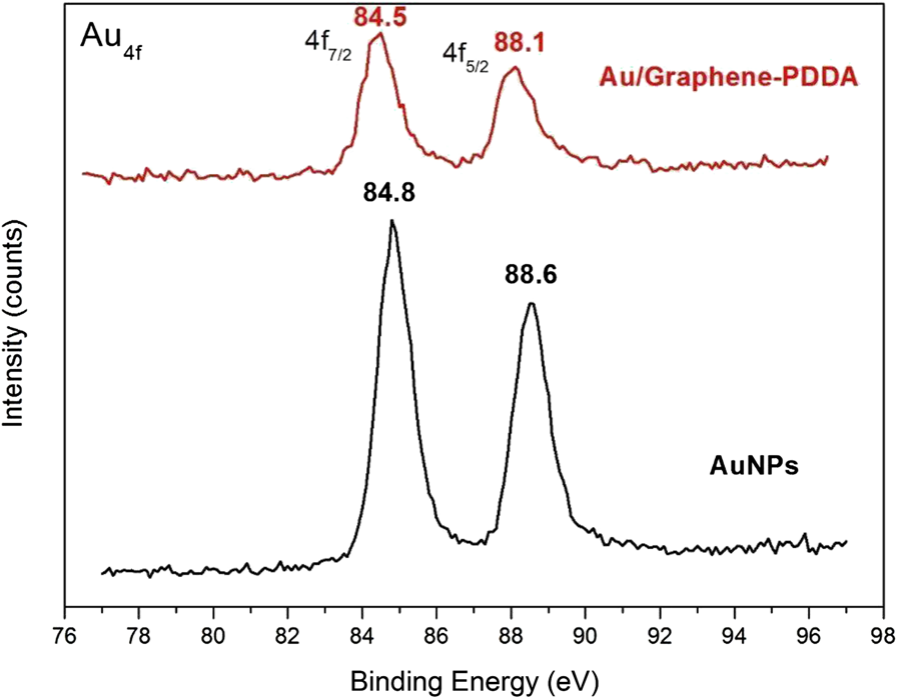
XPS analysis of AuNPs and Au/graphene-PDDA

XPS analysis (Fig. 6) shows that AuNPs bond onto the surface of graphene-PDDA nanosheets, which displays 0.3 and 0.5 eV binding energy shifting in 4f_7/2_ (from 84.8 to 84.5 eV) and 4f_5/2_ (from 88.6 to 88.1 eV), respectively. The binding energy of pristine AuNPs in XPS peaks would decrease after some molecules were grated due to the increase of electrons [21]. It proved that AuNPs truly interacted with graphene-PDDA nanosheets by electrostatic force.

### SERS Application of Au/Graphene-PDDA

**Fig. 7:**
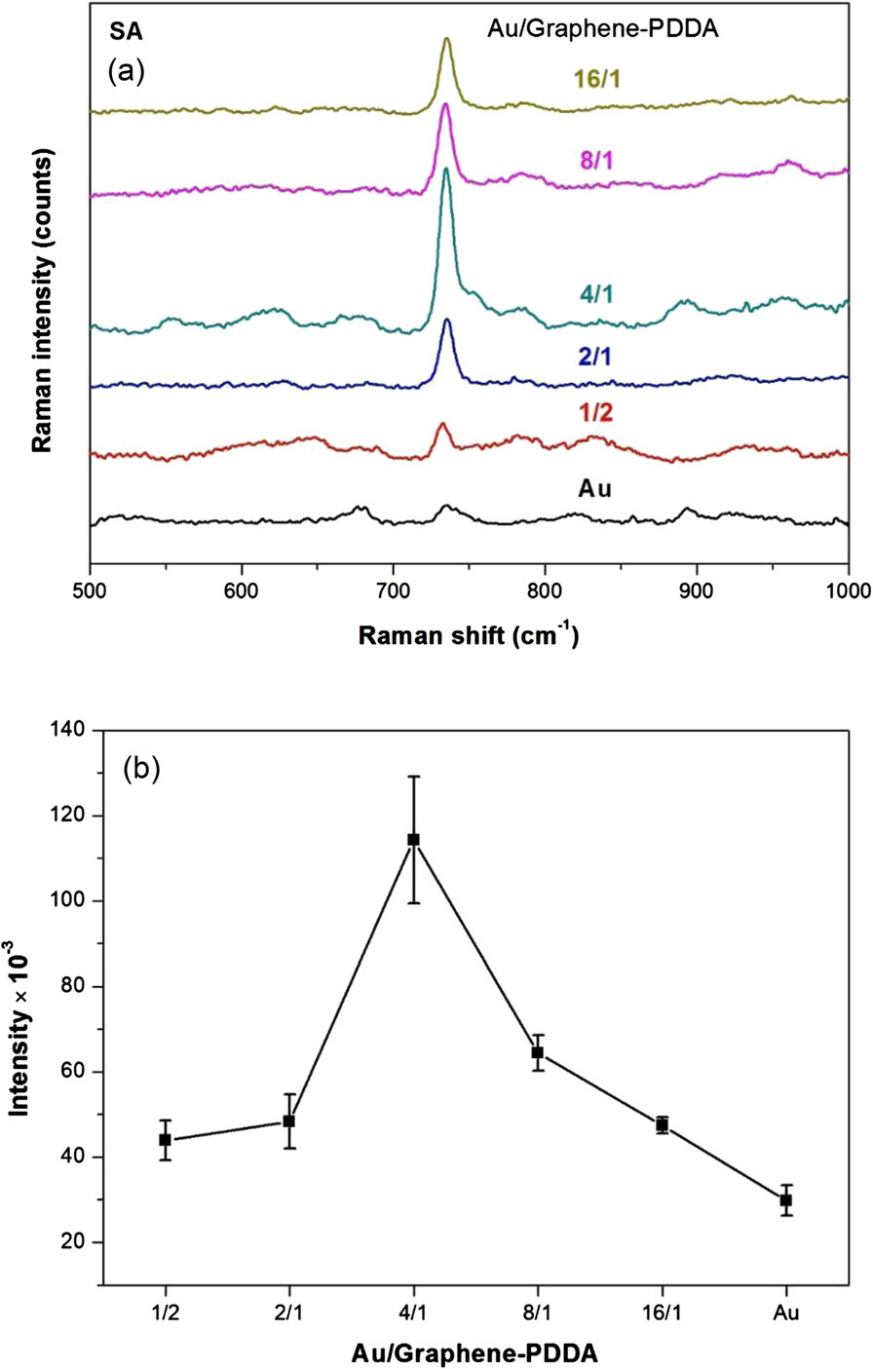
**a** SERS spectra and **b** integrated intensity (733 cm^−1^) of different ratios of Au/graphene-PDDA nanohybrids for bacteria (*S. aureus*) detection

Bacterium (*S. aureus*, SA) was used as a model for SERS detection, and integrated intensity of the Raman signal at 733 cm^−1^ (suggested SERS signal from the cell wall of SA) was examined, as shown in Fig. 7a. Figure 7b and Table 2 illustrate the SERS integrated intensity of *S. aureus* by Au/graphene-PDDA nanohybrids detection. The different ratios of Au/graphene-PDDA nanohybrids were used to detect SA for optimum SERS signal. The results showed that AuNPs/graphene-PDDA in the ratio of AuNPs/graphene-PDDA = 4:1 exhibited the strongest SERS signal in the detection of *S. aureus*.

**Table 2:** Au/graphene-PDDA and their SERS intensity integral of *S. aureus* (integrated range of SERS intensity, 700~770 cm^−1^)

Au/graphene-PDDA	SERS intensity*10^−3^ (integral)
1/2	43.89 ± 4.64
2/1	48.30 ± 6.33
4/1	114.26 ± 14.95
8/1	64.39 ± 4.11
16/1	47.39 ± 1.90
Au	29.75 ± 3.60

**Fig. 8:**
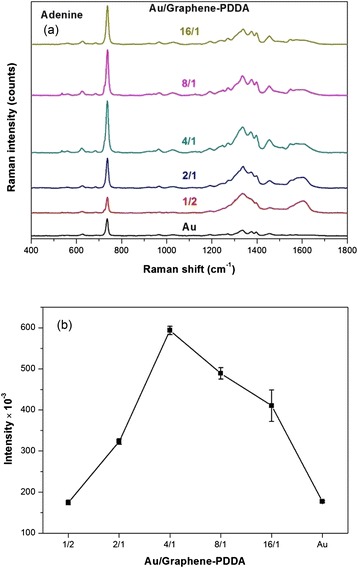
**a** SERS spectra and **b** integrated intensity (733 cm^−1^) of different ratios of Au/graphene-PDDA nanohybrids for small molecules (adenine) detection

In addition, the small molecules (adenine, one component of DNA) also were tested by SERS detection. Figure 8a shows different ratios of AuNPs to graphene-PDDA for adenine SERS detection, and Fig. 8b and Table 3 display that the SERS integrated intensity of adenine by Au/graphene-PDDA nanohybrids detection. The results exhibited that AuNPs/graphene-PDDA in the ratio of AuNPs/graphene-PDDA = 4:1 also illustrated the most optimum SERS signal in the detection of adenine. The ratio of 4:1 (AuNPs/graphene-PDDA) was optimal for higher SERS signal intensity due to the optimal interparticle gaps of AuNPs either in detecting *S. aureus* or adenine. An increase in the ratio of 4:1 would cause the aggregation of AuNPs to induce the laser scattering or decrease the surface plasmon effects (hot junctions effects) due to the contact of AuNPs with each other. Therefore, SERS signal intensity would decrease.

**Table 3:** Au/graphene-PDDA and their SERS intensity integral of adenine (integrated range of SERS intensity, 700~770 cm^−1^)

Au/graphene-PDDA	Intensity*10^−3^ (integral)
1/2	174.76 ± 5.84
2/1	322.91 ± 6.87
4/1	593.43 ± 10.29
8/1	488.96 ± 13.79
16/1	410.37 ± 38.32
Au	177.00 ± 2.04

## Conclusions

This paper demonstrates in detail the synthesis of Au/graphene-PDDA nanocomposites and its application in SERS detection of *S. aureus* and adenine. Graphite was chemically exfoliated, and PDDA was π-π stacked onto the surface of graphene nanosheets, and later, gold nanoparticles were synthesized and attached onto the surface of graphene-PDDA by surface charge interaction. The resulting Au/graphene-PDDA nanocomposites greatly enhanced the Raman signal of *S. aureus* and adenine. Various ratios of AuNPs to graphene-PDDA were tested to make optimum SERS enhancement effects. AuNPs/graphene-PDDA in the ratio of AuNPs/graphene-PDDA = 4:1 exhibited the strongest SERS signal in the bio-detection of small biomolecules (adenine) and microorganisms (*S. aureus*). AuNPs/graphene-PDDA was shown to have enhanced Raman signal capability and unique ability to adsorb onto the microorganisms. Thus, it can be further applied in the rapid and label-free bio-sensing of biomolecules and microorganisms.

## References

[1] Kneipp K, Haka AS, Kneipp H, Badizadegan K, Yoshizawa N, Boone C, Shafer-Peltier KE, Motz JT, Dasari RR, Feld MS (2002). Surface-enhanced Raman Spectroscopy in Single Living Cells using Gold Nanoparticles. Applied Spectroscopy..

[2] Talley CE, Jackson JB, Oubre C, Grady NK, Hollars CW, Lane SM, Huser TR, Nordlander P, Halas NJ (2005). Surface-enhanced Raman scattering from individual Au nanoparticles and nanoparticle dimer substrates. Nano Lett.

[3] Orendorff CJ, Gole A, Sau TK, Murphy CJ (2005). Surface-enhanced Raman spectroscopy of self-assembled monolayers: sandwich architecture and nanoparticle shape dependence. Anal Chem.

[4] Geim AK, Kim P (2008). Graphene, a newly isolated form of carbon, provides a rich lode of novel fundamental physics and practical applications. Sci Am.

[5] Novoselov KS, Geim AK, Morozov SV, Jiang D, Zhang Y, Dubonos SV, Grigorieva IV, Firsov AA (2004). Electric field effect in atomically thin carbon films. Science.

[6] Lee C, Wei X, J. Kysar JW, Hone J (2008). Measurement of the elastic properties and intrinsic strength of monolayer graphene. Science.

[7] Balandin AA, Ghosh S, Bao W, Calizo I, Teweldebrhan D, Miao F, Lau CN (2008). Superior thermal conductivity of single-layer graphene. Nano Lett.

[8] Bolotin KI, Sikes KJ, Jiang Z, Klima M, Fudenberg G, Hone J, Kim P, Stormer HL (2008). Ultrahigh electron mobility in suspended graphene. Solid State Commun.

[9] Rafiee J, Mi X, Gullapalli H, Thomas AV, Yavari F, Shi Y, Ajayan PM, Koratkar NA (2012). Wetting transparency of graphene. Nat Mater.

[10] Chen S, Wu Q, Mishra C, Kang J, Zhang H, Cho K, Cai W, Balandin AA, Ruoff RS (2012). Thermal conductivity of isotopically modified graphene. Nat Mater.

[11] Neto AHC, Novoselov K (2011). Directions in Science and Technology: two-dimensional crystals. Rep Prog Phys.

[12] Novoselov KS, Geim AK, Morozov SV, Jiang D, Katsnelson MI, Grigorieva IV, Dubonos SV, Firsov AA (2005). Two-dimensional gas of massless dirac fermions in graphene. Nature.

[13] Butler GB, Angelo RJ (1957). Preparation and polymerization of unsaturated quaternary ammonium compounds. VIII. A proposed alternating intramolecular-intermolecular chain propagation1. J Am Chem Soc.

[14] Lu J, Wang XD, Xiao CB (2008). Preparation and characterization of konjac glucomannan/poly(diallydimethylammonium chloride) antibacterial blend films. Carbohydr Polym.

[15] Tripathi BP, Dubey NC, Stamm M (2013). Functional polyelectrolyte multilayer membranes for water purification applications. J Hazard Mater.

[16] Yang DQ, Rochette JF, Sacher E (2005). Spectroscopic evidence for pi-pi interaction between poly(diallyl dimethylammonium) chloride and multiwalled carbon nanotubes. J Phys Chem B.

[17] Michaels AM, Jiang, Brus L (2000). Ag nanocrystal junction as the site for surface-enhanced Raman scattering of single rhodamine 6G molecules. J Phys Chem B.

[18] Zhao LL, Jensen L, Schatz GC (2006). Surface-enhanced Raman scattering of pyrazine at the junction between two Ag20 nanoclusters. Nano Lett.

[19] Taton TA, Mirkin CA, Letsinger RL (2000). Scanometric DNA array detection with nanoparticle probe. Science.

[20] Tkachenko AG, Xie H, Coleman D, Glomm W, Ryan J, Anderson MF, Franzen S, Feldheim DL (2003). Multifunctional gold nanoparticle-peptide complexes for nuclear targeting. J Am Chem Soc.

[21] Huang LY, Yang MC (2008). Surface immobilization of chondroitin 6-sulfate/heparin multilayer on stainless steel for developing drug-eluting coronary stents. Colloids Surf B: Biointerfaces.

